# Genome-Wide Identification and Expression Analysis of the bHLH Transcription Factor Family in *Lilium bakerianum* var. *rubrum*

**DOI:** 10.3390/genes16101153

**Published:** 2025-09-28

**Authors:** Zhijia Gu, Mingcheng Wang, Minhui Zhang, Junji Chen, Hongzhi Wu

**Affiliations:** 1 College of Landscape and Horticulture, Yunnan Agricultural University, Kunming 650201, China; guzhijia@mail.kib.ac.cn (Z.G.); zmh97964@163.com (M.Z.); cjj1630724@163.com (J.C.); 2 Key Laboratory of Phytochemistry and Natural Medicines, Kunming Institute of Botany, Chinese Academy of Sciences, Kunming 650201, China; 3 Institute for Advanced Study, Chengdu University, No. 2025 Chengluo Road, Chengdu 610106, China; wangmingcheng@cdu.edu.cn

**Keywords:** *Lilium bakerianum* var. *rubrum*, bHLH transcription factor family, genome-wide identification, gene expression analysis, phylogenetic profiling

## Abstract

Background/Objectives: The basic helix–loop–helix (bHLH) transcription factor family regulates plant development, metabolism, and stress responses. Yet, its genome-wide composition remains unexplored in *Lilium bakerianum* var. *rubrum* (LBVR), an ornamental lily valued for its floral traits. This study aimed to identify, classify, and profile the bHLH family in LBVR using full-length transcriptomic resources. Methods: PacBio HiFi full-length transcriptome sequencing was combined with Illumina RNA-seq for accurate structural annotation and expression quantification. Candidate bHLHs were identified by iTAK and HMMER-Pfam, and their physicochemical properties, secondary structures, motifs, and phylogenetic positions were examined. Expression patterns were analyzed across four floral stages (bud, initial bloom, full bloom, and late bloom). Results: A total of 113 high-confidence bHLH genes were identified, with ~90% successfully annotated. The proteins displayed variation in molecular weight, isoelectric point, structural features, and motif composition. Phylogenetic analysis placed them into 13 clades consistent with *Arabidopsis* subfamilies, revealing lineage-specific expansions and contractions. Expression profiling showed that 95 genes were active in at least one stage, with two transcriptional waves: a strong bud-to-initial-bloom activation and a secondary wave spanning anthesis. Seventeen genes were expressed exclusively at the bud stage, suggesting roles in early floral-organ initiation and pigmentation. Conclusions: This work provides the first genome-wide characterization of bHLHs in LBVR. The integrated sequencing approach generated a robust catalogue and developmental expression map, offering candidates for functional studies and resources for breeding in lilies.

## 1. Introduction

The basic helix–loop–helix (bHLH) transcription factor (TF) family represents one of the largest and most functionally diverse groups of DNA-binding proteins in eukaryotes [1]. Members of this family contain a conserved bHLH domain of approximately 50–60 amino acids (aa), which comprises two amphipathic α-helices separated by a flexible loop region [2]. This structural motif mediates the formation of both the homodimer and heterodimer, as well as specific recognition of E-box (CANNTG) elements in the target gene promoters. The bHLH proteins govern a broad spectrum of developmental and physiological processes in plants, including the regulation of cell proliferation, organ identity, responses to light and phytohormones, adaptation to drought or salinity stresses, and control of flavonoid and alkaloid biosynthetic pathways [3,4]. However, there is a substantial variation in the bHLH gene copy number among several plant species, with over 160 bHLH-encoding loci in *Arabidopsis thaliana* [5], over 180 in rice (*Oryza sativa*) [6], and fewer than 100 loci in basal land plants such as mosses [7]. Further, lineage-specific expansions have given rise to clade-specific bHLH subfamilies that exhibit neo- or sub-functionalization, resulting in both deeply conserved regulators involved in stomatal development and novel factors unique to particular taxa [8,9]. Additionally, functional characterization in model species has shown that certain bHLH clades are indispensable for core processes, while others contribute to species-specific traits, such as pigment accumulation in fruits or defensive metabolite production [10,11]. More recently, transcriptomic analyses have further highlighted the involvement of bHLH transcription factors in regulating plant responses to temperature stress and floral coloration, emphasizing the continued interest in this gene family [12,13]. These observations highlight the evolutionary plasticity of the bHLH family and underscore the value of having a genome-wide catalogue in any given plant species to reveal both conserved modules and unique regulatory innovations.

In the genus *Lilium*, several individual bHLH transcription factors have been reported in recent years. For example, *LpbHLH144* from *Lilium pumilum* enhances salt and alkali stress tolerance when expressed in transgenic tobacco [14]. In *L. oriental* hybrid ‘Siberia’, *LoUDT1* was characterized as a bHLH gene essential for anther development [15]. Moreover, *LvbHLH13* from *L.* ‘Viviana’ was shown to positively regulate anthocyanin accumulation in petals by activating *LvMYB5* [16]. In the same species, *LibHLH22* and *LibHLH63* were identified as positive regulators of volatile terpenoid biosynthesis, directly enhancing the expression of key terpene pathway genes [17]. Beyond bHLH factors, other transcriptional regulators have also been implicated in lily traits: for instance, *LhMYB114* together with structural genes such as *LhDFR* and *LhANS-rr1* regulates anthocyanin biosynthesis in flower buds of *L*. ‘Siberia’ [18], while transcriptomic studies in LA lily ‘Aladdin’ revealed that transcription factors including BLHs, ARFs, HD-ZIPs, AP2/ERFs, and SBPs are involved in hormonal and sugar-mediated control of stem bulblet formation [19]. Collectively, these studies highlight the importance of transcription factors, particularly bHLHs, in controlling developmental processes, pigment biosynthesis, secondary metabolism, and stress responses in lilies. However, most reports have focused on single-gene functional analyses or specific physiological pathways, and no genome-wide investigation of the bHLH gene family has yet been reported in lilies.

*L. bakerianum* var. *rubrum* (LBVR) is an ornamental lily species endemic to the montane regions of Yunnan Province in Southwestern China [20] distinguished by its vibrant magenta-red petals, strong floral fragrance, and graceful floral architecture, which also make it highly prized in both commercial horticulture and traditional ornamental gardens. Besides its aesthetic appeal, LBVR is also utilized in ethnobotanical applications due to its anti-inflammatory and antioxidant properties. However, despite its economic, ecological, and cultural significance, no genome-wide survey of TFs has been conducted in this species. Therefore, a systematic characterization of the bHLH family in LBVR is critical to uncover the regulators underlying its unique floral traits, pigment biosynthesis, scent production, and stress resilience. Moreover, understanding the repertoire and expression dynamics of bHLH genes may facilitate targeted breeding strategies aimed at enhancing flower color intensity, extending vase life and improving tolerance to abiotic stresses such as temperature fluctuations encountered during commercial cultivation and postharvest storage.

The advent of high-throughput sequencing technologies, including combined transcriptomics and third-generation sequencing, provides powerful tools for mining genes involved in secondary metabolism in both medicinal and ornamental plants [21]. In this study, we conducted a genome-wide identification and characterization of bHLH TFs in LBVR using high-quality transcriptome data from floral tissues at various developmental stages. By integrating homology-based prediction, hidden Markov model (HMM)-based domain searches, conserved domain validation, protein property assessment, motif composition, gene structure, phylogenetic relationships, and expression profiling, we identified a high-confidence bHLH gene set. This work provides new insights into the regulatory roles of bHLH TFs in floral development and specialized metabolism and also offers a valuable resource for future functional and breeding studies in LBVR and related ornamental species.

## 2. Materials and Methods

### 2.1. Full-Length Transcriptome Sequencing and Annotation

The plant materials used for sequencing in this study were collected from a mature individual of LBVR growing on Changchong Mountain in Kunming, Yunnan Province, Southwestern China (25.1177° N, 102.7079° E). Floral tissues at four developmental stages, namely, bud, initial bloom, full bloom, and late bloom (Figure S1), were collected and promptly frozen in liquid nitrogen to prevent RNA degradation. High-quality RNA was extracted from the pooled samples. Following quality control assessments of purity, concentration, and integrity, full-length cDNA was synthesized from the mRNA and subsequently amplified by PCR. The resulting cDNA was then subjected to damage- and end-repairs. SMRTbell adapters were ligated to the repaired cDNA to construct SMRTbell template libraries. High-fidelity (HiFi) long reads were generated through single-molecule sequencing on the PacBio Revio platform (Pacific Biosciences, Menlo Park, CA, USA).

High-accuracy circular consensus sequences (CCSs) were generated from subreads using the CCS tool in SMRT Link v10.1 (Pacific Biosciences, Menlo Park, CA, USA), with a minimum of three full passes and a read quality threshold of ≥0.9. The resulting CCS reads were then classified as full- or non-full-length transcripts based on the presence of intact 5′ primers, 3′ primers, and poly(A) tails. FLNC sequences were processed using the Iso-Seq module in SMRT Link to cluster similar sequences into distinct groups, with each cluster subsequently collapsed into a single consensus isoform. Redundant isoforms were then removed using CD-HIT v4.6.1 [22] with a 99% sequence identity threshold. The completeness of the non-redundant transcripts was subsequently assessed using BUSCO v3.0.2 [23], based on the OrthoDB database of lineage-specific single-copy orthologs.

The coding sequences (CDSs) of the assembled transcripts were predicted using TransDecoder v5.0.0 [24]. Functional annotation was performed using DIAMOND v2.0.15 [25] by aligning the predicted protein sequences against multiple databases, including the NCBI Non-Redundant Protein Sequence Database (NR) [26], Swiss-Prot, TrEMBL [27], eggNOG [28], Clusters of Orthologous Groups of proteins (COG) [29], Eukaryotic Orthologous Groups (KOG) [30], and the Kyoto Encyclopedia of Genes and Genomes (KEGG) [31], with an E-value cutoff of <1 × 10^−5^ Conserved protein domains and Gene Ontology (GO) annotations were obtained using InterProScan v5.34-73.0 [32].

### 2.2. Identification of bHLH TFs

Candidate bHLH TFs were identified by integrating iTAK prediction with Pfam domain validation. Putative bHLH genes were first predicted using iTAK v1.7a [33] with default plant-specific parameters and then screened for the conserved bHLH domain (PF00010) using HMMER v3.3.2 [34] with an E-value cutoff of <1 × 10^−5^. The final candidates were defined as bHLH transcripts after being identified by both methods.

The physicochemical properties of the identified bHLH proteins, including sequence length, molecular weight, isoelectric point, instability index, and GRAVY, were calculated using the ProteinAnalysis module from Biopython’s ProtParam package based on their amino acid sequences. Further, the subcellular localization of the bHLH proteins was examined using DeepLoc-2.0 [35], which is a deep learning (DL)-based predictor trained on eukaryotic proteins, while their secondary structures were predicted using NetSurfP-3.0 [36], which estimates the probabilities of α-helix, β-strand, and coil structures using DL models. Conserved motif analysis of the bHLH proteins was conducted using MEME Suite v5.5.8 [37] in the classical mode. The number of motifs was set to 15, with widths ranging from 6 to 50 aa, while the site distribution was set to zero or one occurrence per sequence.

### 2.3. Phylogenetic Analysis and Classification of the bHLH TFs

A total of 153 *A. thaliana* bHLH protein sequences with the longest isoforms for each gene were downloaded from PlantTFDB 4.0 [38] and combined with 113 LBVR bHLH proteins identified in this study. Multiple sequence alignment was used to align the protein sequences using MAFFT v7.525 [39] with the linsi mode for high-accuracy alignment. The resulting alignment was trimmed using trimAl v1.4 [40] with the automated1 mode to remove poorly aligned and divergent regions. Further, ML phylogenetic inference was conducted using RAxML v8.2.12 [41] with the PROTGAMMAILGX model with 500 replicates for Bootstrap using the rapid bootstrapping algorithm.

### 2.4. Transcriptome Sequencing and Expression Level Analysis

Floral tissues of LBVR were collected at bud (ST1), initial bloom (ST2), full bloom (ST3), and late bloom (ST4) developmental stages, with three biological replicates per stage. All samples were immediately frozen in liquid nitrogen and stored at −80 °C to prevent RNA degradation until RNA extraction. Total RNA was extracted using TRIzol reagent (Invitrogen, Carlsbad, CA, USA), and high-quality RNA samples were used for library preparation. mRNA was enriched from total RNA using oligo(dT) magnetic beads and converted into cDNA libraries using the TruSeq Stranded mRNA Library Prep Kit (Illumina, San Diego, CA, USA) according to the manufacturer’s protocol. The libraries were then sequenced using the DNBSEQ-T7 platform (MGI Tech, Shenzhen, China) to generate 150 bp paired-end reads. Raw sequencing reads were quality-filtered using Trimmomatic v0.39 [42]. Clean reads were subsequently aligned to the full-length transcriptome reference using Bowtie2 v2.5.4 [43], while the expression levels of each transcript were quantified as FPKM (fragments per kilobase of transcript per million mapped reads) using StringTie v1.3.3 [44]. Transcript-level count data were obtained using StringTie and aggregated into count matrices via the prepDE.py script for downstream differential gene expression analysis. Differential expression between successive floral stages, including ST1 vs. ST2, ST2 vs. ST3, and ST3 vs. ST4, was analyzed in DESeq2 (Bioconductor), with genes showing |log2 fold change| > 1 and Benjamini–Hochberg-adjusted *p* < 0.05 classified as differentially expressed genes (DEGs).

## 3. Results

### 3.1. Summary of Transcriptome Assembly and Annotation

**Figure 1:**
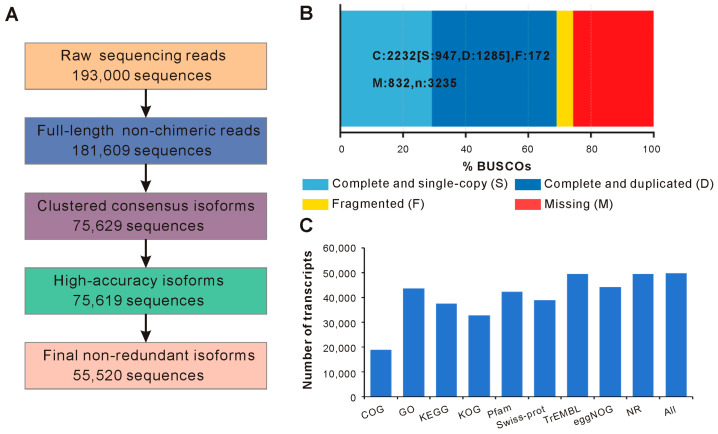
Summary of transcriptome assembly and annotation. (**A**) Workflow illustrating the processing steps of PacBio HiFi data. (**B**) BUSCO evaluation of the completeness of the transcriptome based on the conserved single-copy orthologs. (**C**) Bar chart showing the number of transcript isoforms annotated in different functional databases.

A total of 193,000 CCSs were generated, yielding approximately 409 million bases (Mb) of HiFi data with an average read length of 2120 bp. Among these CCSs, 181,609 (94.10%) were identified as full-length non-chimeric (FLNC) sequences, indicating high-quality transcript capture. Similar sequences were then clustered into 75,629 consensus isoforms with an average length of 1858 bp, of which 75,619 exhibited high accuracy (>99%). After removing redundancy, a final set of 55,520 non-redundant transcript isoforms was obtained (Figure 1A). BUSCO analysis yielded a completeness score of 68.97%, indicating moderate representation of conserved single-copy orthologs in the assembled transcriptome (Figure 1B). Coding sequence prediction resulted in 52,216 open reading frames (ORFs), among which 29,660 were classified as complete. The functional annotation results showed that 49,810 transcript isoforms (89.72%) were successfully annotated in at least one database (Figure 1C). Among them, 43,700 (78.71%) were assigned Gene Ontology (GO) terms, and 42,304 (76.20%) contained identifiable Pfam domains.

### 3.2. Identification and Features of bHLH TFs

**Figure 2:**
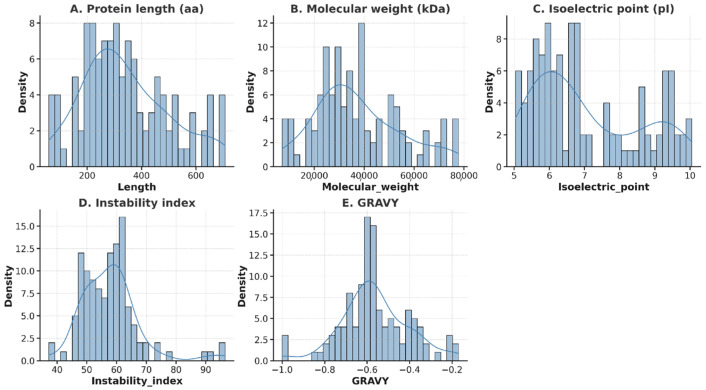
Physicochemical properties of bHLH proteins. Each panel shows a histogram overlaid with a kernel density estimation curve, representing the frequency and overall distribution of the respective property across all identified bHLH proteins. (**A**) Protein length. (**B**) Molecular weight. (**C**) Isoelectric point. (**D**) Instability index. (**E**) Grand average of hydropathicity.

A total of 4388 TFs were predicted in LBVR using iTAK, out of which 113 were identified as bHLH TFs, consistent with domain-based annotation, which also detected 113 transcripts containing the conserved bHLH domain. The overlap of both methods also yielded 113 high-confidence bHLH TFs. The lengths of the identified bHLH proteins ranged from 59 to 707 aa (Figure 2A), with corresponding molecular weights of between 7.15 and 77.78 kDa (Figure 2B). The predicted isoelectric points of the bHLH TFs varied from 5.04 to 10.05 (Figure 2C), while their instability index values ranged from 37.23 to 96.46 (Figure 2D). Furthermore, all proteins exhibited negative grand average of hydropathicity (GRAVY) values, ranging from −1.00 to −0.17 (Figure 2E), indicating that they are generally hydrophilic.

A total of 111 bHLH proteins were found exclusively in the nucleus, while baihe_transcript_30752 and baihe_transcript_55079 were localized to either the cytoplasm or the nucleus. Secondary structure prediction indicated that the bHLH proteins predominantly contain coil regions (78.41%), followed by α-helices (18.94%) and a small proportion of β-strands (2.65%), which is consistent with the expected helix–loop–helix (HLH) motif, suggesting considerable structural flexibility of the bHLH proteins. In addition to the conserved HLH domain (PF00010), domain composition analysis revealed that seven bHLH proteins contained an N-terminal domain specific to bHLH-MYC and R2R3-MYB TFs (PF14215), suggesting potential functional diversification. Interestingly, protein baihe_transcript_54573 had two tandem PF00010 domains, indicating possible internal duplication. These multi-domain configurations imply that certain bHLH members may participate in broader or specialized regulatory pathways.

**Figure 3:**
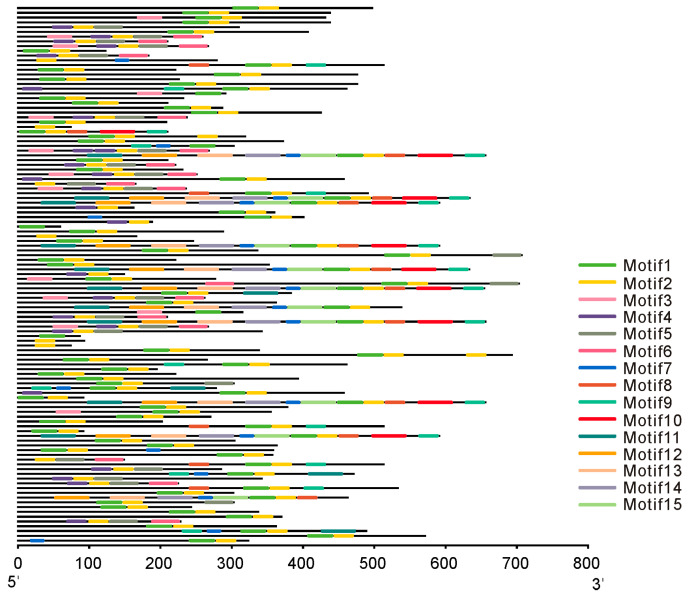
Distribution of conserved motifs in bHLH transcription factors. Each horizontal bar represents a bHLH protein, with colored boxes indicating the positions and identities of up to 15 predicted motifs. The full list of gene IDs is provided in Table S1.

To further explore the structural diversity of the bHLH protein family, a total of 15 distinct motifs were identified, with lengths ranging from 21 to 50 aa (Figure 3). Of these, Motif 1 was present in 81 of the 113 bHLH proteins with the highest E-value of 1.5 × 10^−1699^, corresponding well with the canonical HLH DNA-binding domain. However, Motif 2 was the most frequently occurring motif, with occurrence in 107 sequences, suggesting that it may represent a highly conserved auxiliary region shared by most bHLH members. The diversity in motif presence, order, and combination patterns across bHLH proteins reflects both the conserved nature of the HLH core and the evolutionary divergence in their flanking regions, which may contribute to differences in DNA-binding specificity or regulatory interactions.

### 3.3. Subfamily Classification of bHLH Genes

**Figure 4:**
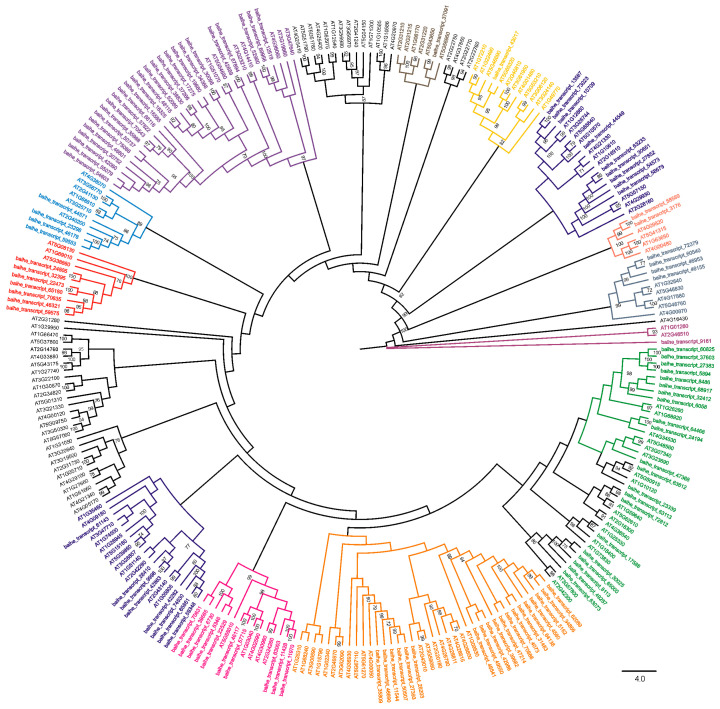
Maximum likelihood phylogenetic tree of bHLH transcription factors from *L. bakerianum* var. *rubrum* and *A. thaliana*. Bootstrap support values (>70%) from 1000 replicates are indicated at the corresponding nodes.

To clarify the evolutionary relationships and potential functions of bHLH genes, a maximum likelihood (ML) phylogenetic tree was constructed based on sequence alignments of bHLH genes from *A. thaliana* and LBVR. The bHLH genes in LBVR clustered into 13 distinct clades (Figure 4). In one clade, the gene *baihe_transcript_43017* was most closely related to *AT5G65320* and clustered with *AT1G49770*, which is a well-characterized bHLH TF known to regulate embryo development in *A. thaliana* [45]. However, in the largest clade, 21 LBVR genes clustered with 17 *A. thaliana* bHLH genes, several of which have well-established roles, including *AT1G68920*, which is involved in flowering-time regulation [46]; *AT1G25330*, which is involved in transmitting tract development [47]; and *AT1G73830*, which positively regulates shade avoidance [48]. Interestingly, several *A. thaliana* bHLH genes appeared as singleton branches in the phylogenetic tree, without clustering with any LBVR homologs.

### 3.4. Expression Patterns of bHLH Genes

**Figure 5:**
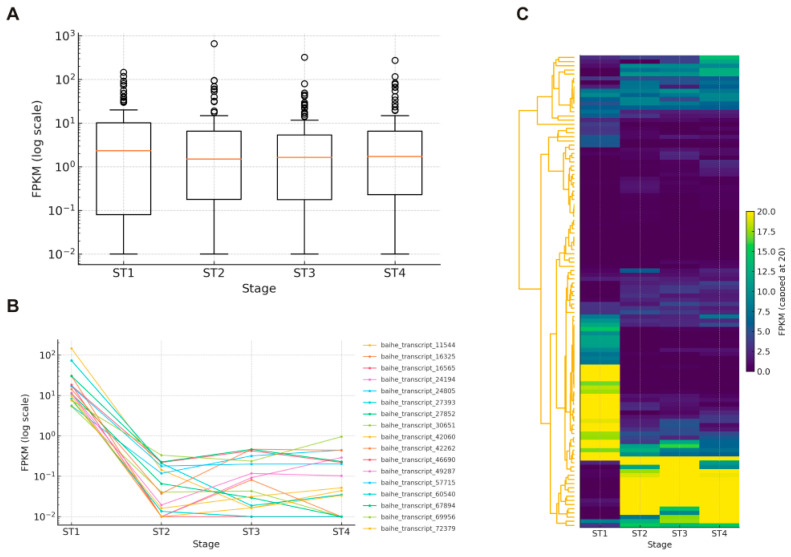
Expression patterns of bHLH genes in *L. bakerianum* var. *rubrum* across four floral stages. (**A**) Boxplots of log10-FPKM for all 113 genes were generated using three biological replicates per stage. (**B**) Temporal profiles of 17 ST1-specific genes with FPKM ≥ 5 only at the ST1 stage. (**C**) Heat-map with hierarchical clustering revealing three expression clusters, namely, cluster 1 with uniformly low expression, cluster 2 with ST1-biased expression, and cluster 3 with ST2-ST4-induced expression.

To explore the potential roles of bHLH genes during floral development in LBVR, we analyzed their expression patterns across four developmental stages, namely, ST1, ST2, ST3, and ST4. Transcript abundance of the identified bHLH genes was quantified using FPKM, while distribution profiling, stage-specific expression screening, expression clustering, and pairwise differential expression analysis were performed between consecutive floral stages. Of the 113 bHLH genes, 95 (84.07%) exhibited an average FPKM > 1 in at least one developmental stage, suggesting their widespread transcriptional activity during floral development. On average, the expression levels of the bHLH genes were highest in the ST2 (13.52) and ST1 (11.40) stages, suggesting early transcriptional activation before and during anthesis (Figure 5A). However, the bHLH gene expression declined slightly at ST3 (9.12) but moderately increased at ST4 (10.59). Therefore, the transient decrease at ST3 may reflect a regulatory shift associated with peak anthesis.

Stage-specific expression screening was used to identify bHLH genes that were highly expressed at only one floral stage. Genes with an average FPKM ≥ 5 at exactly one developmental stage but with FPKM < 1 at all other stages were defined as stage-specific. Thus, based on this criterion, 17 bHLH genes were specifically expressed at ST1 and none at ST2, ST3, or ST4 (Figure 5B). These ST1-specific genes are likely involved in early floral-organ initiation and the developmental transition toward anthesis. Further, hierarchical clustering of the four-stage matrix resolved three expression modules (Figure 5C). For instance, cluster 1 consisted of 74 genes (65.48%) with low-to-moderate and uniform expression; cluster 2, with 22 genes (19.47%), was strongly ST1-biased, mirroring the stage-specific set; while cluster 3, containing 16 genes (14.16%), was up-regulated from ST2 onward, marking anthesis progression and later maturation. However, one highly expressed gene formed an outlier cluster. Therefore, these results point to two major expression waves, consisting of an early burst at the ST1 stage and a later activation spanning the ST2-ST4 stages, through which bHLH TFs coordinate successive phases of LBVR flower development.

**Figure 6:**
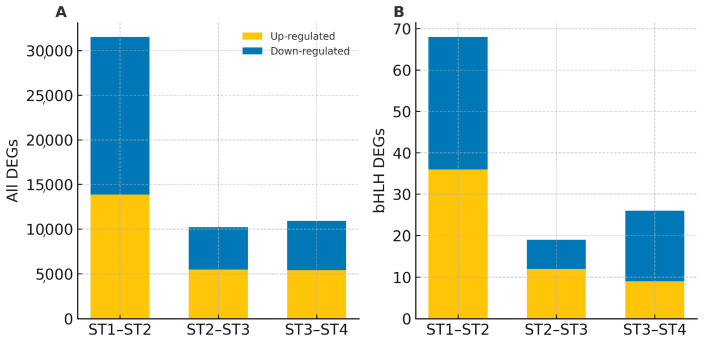
Stage-to-stage differentially expressed genes (DEGs) in *L. bakerianum* var. *rubrum*. Stacked bars show up-regulated (yellow) and down-regulated (blue) genes. (**A**) shows whole transcriptome and (**B**) the bHLH subset transcriptome.

Pairwise differential gene expression analysis revealed two major transcriptional waves that parallel the expression profiles of bHLH TFs. The first transcriptional wave from ST1 to ST2 involved 31,536 DEGs, with 13,866 genes up-regulated and 17,670 down-regulated (Figure 6A). However, subsequent transitions were smaller, with 10,190 genes changed between ST2 and ST3 and 10,899 genes differentially expressed between ST3 and ST4. Interestingly, the expression of bHLH genes also followed the same trend (Figure 6B). For instance, 68 of the 113 bHLH family members were differentially expressed in the transition between ST1 and ST2, with 36 up-regulated and 32 down-regulated. On the other hand, only 19 DEGs responded in the ST2–ST3 comparisons, with 12 up-regulated and 7 down-regulated, while 26 DEGs were expressed in the ST3–ST4 transitions, with 9 up-regulated and 17 down-regulated. Therefore, the majority of bHLH reconfiguration accompanies the large-scale rewiring that initiates floral-organ formation at the bud and initial bloom stages, whereas later stages of full and late blooms require progressively fewer bHLH adjustments to fine-tune anthesis progression and floral maturation.

## 4. Discussion

Unlike conventional short-read RNA-seq, full-length transcriptome sequencing with PacBio HiFi reads captures complete cDNA molecules and reduces assembly artefacts, which is particularly valuable for gene family studies. In this study, we combined PacBio long reads with Illumina short reads to achieve both accurate structural annotation and reliable expression quantification of bHLH genes in LBVR. Two independent pipelines consistently identified 113 bHLH genes, providing a robust catalogue despite the organ-specific bias of the floral transcriptome libraries. This integrated approach not only maximized confidence in gene models but also enabled stage-resolved expression profiling across floral development, thereby establishing a solid foundation for functional studies.

The bHLH family is one of the largest and most heterogeneous TF lineages in plants [3,4]. In LBVR, the 113 identified bHLHs represent a smaller repertoire compared with representative monocots like rice (~180) [6], maize (>200) [49], and wheat (>470) [50] but are closer to numbers reported for other ornamentals such as *Dendrobium officinale* (98) [51] and *Cymbidium ensifolium* (94) [52]. This suggests lineage-specific contraction of the family in lilies, which may reflect functional consolidation or unique adaptive pressures associated with perennial growth and ornamental traits. Phylogenetic analysis placed the LBVR bHLH proteins into 13 clades, largely consistent with the canonical *Arabidopsis* subfamilies, highlighting both evolutionary conservation and divergence. For instance, the clade that contained the four *Arabidopsis* flavonoid-regulatory paralogues TT8 (*AT4G09820*), GL3 (*AT5G41315*), EGL3 (*AT1G63650*), and MYC1 (*AT1G32640*) was represented in LBVR by only two orthologous genes, indicating a lineage-specific contraction relative to *Arabidopsis*. Conversely, the clade housing flowering-time, transmitting-tract, and shade-avoidance regulators, such as *AT1G68920*, *AT1G25330*, and *AT1G73830*, contain 21 LBVR paralogues compared to 17 *Arabidopsis* genes, indicating a lineage-specific expansion. Therefore, phylogenetic profiling provides a framework that prioritizes bHLH candidates for functional assays, as has been proven in previous studies [53,54,55].

Structural analyses further support this view. LBVR bHLHs displayed wide variation in molecular weight, isoelectric point, secondary-structure content, and motif organization. Such diversity parallels findings in other monocots and *Arabidopsis*, where subfamily-specific motifs often correlate with specialized roles in developmental or metabolic pathways [49,51,56]. The structural heterogeneity observed in LBVR thus provides a molecular basis for potential functional divergence, particularly in processes unique to lilies, such as volatile biosynthesis and bulb development.

Expression profiling refined these evolutionary insights by highlighting functional candidates. Among the 113 genes, 95 were actively expressed in at least one floral stage, with two major transcriptional waves: a strong activation at the early bud stage and a secondary wave spanning initial to late bloom. Notably, 17 genes were exclusively expressed at the bud stage, suggesting critical roles in early floral-organ initiation. This early-stage bias contrasts with rice, where some bHLHs peak around anthesis [6,57,58], but is consistent with observations in other lilies, where bHLHs regulate pigmentation and stress responses at pre-anthesis stages [14,16,18]. Moreover, the majority of transcriptomic reprogramming occurred during the bud-to-initial-bloom transition, whereas later stages involved fewer adjustments, pointing to an early commitment of transcriptional regulation in LBVR floral development.

Overall, the contraction of the bHLH family size, the enrichment of certain clades, and the strong early-stage transcriptional activation distinguish LBVR from both cereals and *Arabidopsis*. These findings suggest that lilies may rely on fewer but potentially more specialized bHLH regulators to coordinate floral development and ornamental traits, highlighting both evolutionary streamlining and adaptive specialization.

## 5. Conclusions

This study presents the first genome-wide characterization of the bHLH transcription factor family in LBVR. A total of 113 high-confidence bHLH genes were identified, fewer than in other monocots. Phylogenetic analysis grouped them into 13 distinct clades, reflecting both conserved regulators and lineage-specific variation. The bHLH proteins also showed high diversity in physicochemical traits, secondary structures, and MEME motifs, suggesting potential functional diversification. Expression profiling across four floral stages revealed two major transcriptional waves, with 17 genes specifically activated at the early floral bud stage, indicating roles in floral-organ initiation. These findings provide candidate regulators for floral development, pigmentation, and stress responses and offer a valuable resource for future functional and breeding studies in lilies.

## Data Availability

The transcriptome assembly and annotation have been deposited in Figshare under the DOI https://doi.org/10.6084/m9.figshare.30226042.

## Supplementary Materials

The following supporting information can be downloaded at: https://www.mdpi.com/article/10.3390/genes16101153/s1, Figure S1. Representative photographs of *L. bakerianum* var. *rubrum* flowers at different developmental stages used for transcriptomic analysis. Table S1, List of bHLH transcription factor gene IDs in the same order as presented in Figure 3.

## Abbreviations

The following abbreviations are used in this manuscript:

bHLH	Basic Helix–Loop–Helix
LBVR	*L. bakerianum* var. *rubrum*
TF	Transcription Factor
HMM	Hidden Markov Model
aa	Amino Acids
GRAVY	Grand Average of Hydropathicity
ML	Maximum Likelihood
FPKM	Fragments per Kilobase of Transcript per Million Mapped Reads
DEGs	Differentially Expressed Genes
ORFs	Open Reading Frames
BUSCO	Benchmarking Universal Single-Copy Orthologs
