# Supplementary material for: Genome-Wide Identification and Expression Analysis of the bHLH Transcription Factor Family in Lilium bakerianum var. rubrum
Source: Genes (Basel). 2025 Sep 28;16(10):1153. doi: 10.3390/genes16101153 (PMC12563917; doi:10.3390/genes16101153)
Supplement: Supplementary file 1 [file genes-16-01153-s001.zip › genes-3879861-supplementary.docx]

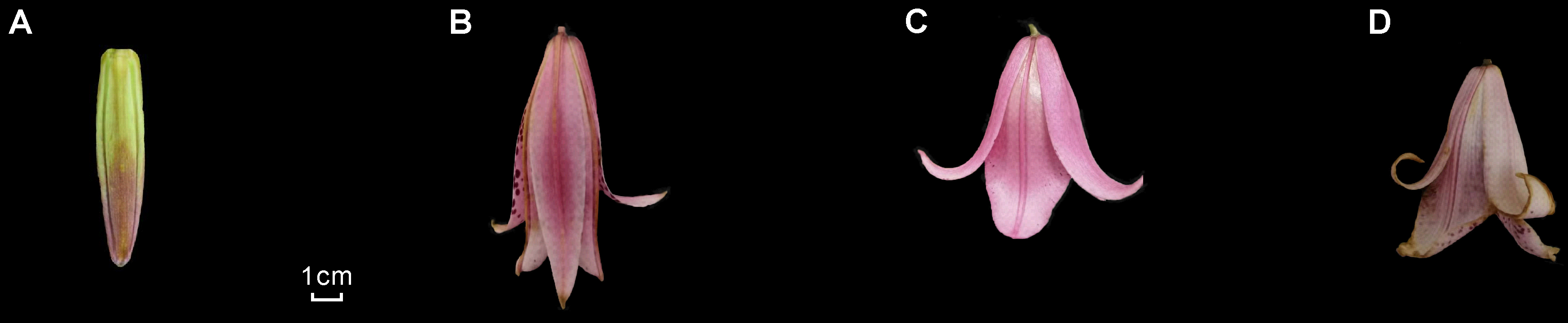


**Figure S1.** Representative photographs of *Lilium bakerianum* var. *rubrum* flowers at different developmental stages used for transcriptomic analysis. (A) Bud stage; (B) Initial bloom; (C) Full bloom; (D) Late bloom. A common scale bar is provided and applies to all panels.

**Table S1**. List of bHLH transcription factor gene IDs in the same order as presented in Figure 3.

| baihe_transcript_10709 |
| --- |
| baihe_transcript_11428 |
| baihe_transcript_11544 |
| baihe_transcript_11970 |
| baihe_transcript_12519 |
| baihe_transcript_13587 |
| baihe_transcript_16325 |
| baihe_transcript_16565 |
| baihe_transcript_17275 |
| baihe_transcript_17588 |
| baihe_transcript_19680 |
| baihe_transcript_22473 |
| baihe_transcript_22529 |
| baihe_transcript_23298 |
| baihe_transcript_23339 |
| baihe_transcript_24194 |
| baihe_transcript_24805 |
| baihe_transcript_27383 |
| baihe_transcript_27393 |
| baihe_transcript_27852 |
| baihe_transcript_28203 |
| baihe_transcript_28410 |
| baihe_transcript_30025 |
| baihe_transcript_30098 |
| baihe_transcript_30651 |
| baihe_transcript_30752 |
| baihe_transcript_31483 |
| baihe_transcript_3176 |
| baihe_transcript_32395 |
| baihe_transcript_32412 |
| baihe_transcript_34368 |
| baihe_transcript_34866 |
| baihe_transcript_35809 |
| baihe_transcript_35968 |
| baihe_transcript_37091 |
| baihe_transcript_37266 |
| baihe_transcript_37603 |
| baihe_transcript_37822 |
| baihe_transcript_38830 |
| baihe_transcript_39565 |
| baihe_transcript_39682 |
| baihe_transcript_4073 |
| baihe_transcript_42060 |
| baihe_transcript_42262 |
| baihe_transcript_42863 |
| baihe_transcript_42889 |
| baihe_transcript_42986 |
| baihe_transcript_43017 |
| baihe_transcript_44549 |
| baihe_transcript_44571 |
| baihe_transcript_4580 |
| baihe_transcript_46117 |
| baihe_transcript_46155 |
| baihe_transcript_46178 |
| baihe_transcript_46321 |
| baihe_transcript_46541 |
| baihe_transcript_46601 |
| baihe_transcript_46690 |
| baihe_transcript_46953 |
| baihe_transcript_47214 |
| baihe_transcript_47368 |
| baihe_transcript_48115 |
| baihe_transcript_49287 |
| baihe_transcript_49550 |
| baihe_transcript_50507 |
| baihe_transcript_50737 |
| baihe_transcript_5162 |
| baihe_transcript_52069 |
| baihe_transcript_52960 |
| baihe_transcript_54573 |
| baihe_transcript_54603 |
| baihe_transcript_55079 |
| baihe_transcript_57715 |
| baihe_transcript_58589 |
| baihe_transcript_58679 |
| baihe_transcript_5894 |
| baihe_transcript_59348 |
| baihe_transcript_59553 |
| baihe_transcript_59575 |
| baihe_transcript_60540 |
| baihe_transcript_6058 |
| baihe_transcript_60825 |
| baihe_transcript_61143 |
| baihe_transcript_62089 |
| baihe_transcript_63003 |
| baihe_transcript_63073 |
| baihe_transcript_63113 |
| baihe_transcript_63233 |
| baihe_transcript_6346 |
| baihe_transcript_63612 |
| baihe_transcript_64138 |
| baihe_transcript_64468 |
| baihe_transcript_65000 |
| baihe_transcript_65180 |
| baihe_transcript_65851 |
| baihe_transcript_66116 |
| baihe_transcript_6730 |
| baihe_transcript_67894 |
| baihe_transcript_68917 |
| baihe_transcript_69956 |
| baihe_transcript_70543 |
| baihe_transcript_70631 |
| baihe_transcript_70835 |
| baihe_transcript_70888 |
| baihe_transcript_72379 |
| baihe_transcript_72812 |
| baihe_transcript_73023 |
| baihe_transcript_74530 |
| baihe_transcript_75088 |
| baihe_transcript_8113 |
| baihe_transcript_8486 |
| baihe_transcript_9161 |
| baihe_transcript_9998 |
